# Characterization of *Burkholderia pseudomallei* protein BPSL1375 validates the Putative hemolytic activity of the COG3176 N-Acyltransferase family

**DOI:** 10.1186/s12866-015-0604-4

**Published:** 2015-11-23

**Authors:** Laziana Ahmad, Teng Loong Hung, Nor Azurah Mat Akhir, Rahmah Mohamed, Sheila Nathan, Mohd Firdaus-Raih

**Affiliations:** School of Biosciences and Biotechnology, Faculty of Science and Technology, Universiti Kebangsaan Malaysia, 43600 UKM, Bangi, Selangor Malaysia; Malaysia Genome Institute, 43000, Kajang, Selangor Malaysia; INTI International University, Bandar Baru Nilai, Nilai, Negeri Sembilan 71800 Malaysia; Institute of Systems Biology, Universiti Kebangsaan Malaysia, 43600 UKM, Bangi, Selangor Malaysia

**Keywords:** *Burkholderia pseudomallei*, Hemolysin, BPSL1375, N-acyltransferase

## Abstract

**Background:**

There are still numerous protein subfamilies within families and superfamilies that do not yet have conclusive empirical experimental evidence providing a specific function. These proteins persist in databases with the annotation of a specific ‘putative’ function made by association with discernible features in the protein sequence.

**Results:**

Here, we report the characterization of one such protein produced by the pathogenic soil bacterium *Burkholderia pseudomallei*, BPSL1375, which provided evidence for putative hemolysins in the COG3176 family to have experimentally validated hemolytic activity. BPSL1375 can be classified into the N-acyltransferase superfamily, specifically to members of the COG3176 family. Sequence alignments identified seven highly conserved residues (Arg54, Phe58, Asp75, Asp78, Arg99, Glu132 and Arg135), of which several have been implicated with N-acyltransferase activity in previously characterized examples. Using the 3D model of an N-acyltransferase example as a reference, an acyl homoserine lactone synthase, we generated 3D structure models for mutants of six of the seven N-acyltransferase conserved residues (R54, D75, D78, R99, E132 and R135). Both the R99 and R135 mutants resulted in a loss of hemolytic activity while mutations at the other five positions resulted in either reduction or increment in hemolytic activity.

**Conclusions:**

The implication of residues previously characterized to be important for N-acyltransferase activity to hemolytic activity for the COG3176 family members of the N-acyltransferase provides validation of the correct placement of the hemolytic capability annotation within the N-acyltransferase superfamily.

**Electronic supplementary material:**

The online version of this article (doi:10.1186/s12866-015-0604-4) contains supplementary material, which is available to authorized users.

## Background

*Burkholderia pseudomallei* is a soil bacterium that causes melioidosis, a deadly disease endemic to South East Asia and Northern Australia [1]. This bacterium is recognized as a potential category B biothreat agent due to its aerosol infectivity and severe course of infection [2]. Pathogenesis of *B. pseudomallei* remains poorly understood despite a number of previous studies having described the expression of several virulence factors, particularly extracellular products with proteolytic, lipolytic and hemolytic activity [3, 4], the Type VI secretion system [5] and the capsule [6]. A toxin that deamidates and subsequently inactivates helicase activity of the translation initiation factor (eIF4a) has been reported [7]. Other aspects of this bacterium’s genome has also been explored including the existence of small RNAs [8] and methylation of genomic DNA by restriction modification systems [9]. Nevertheless, much of the genome remains annotated as genes encoding hypothetical proteins.

Most bacterial pathogens have the capacity to acquire nutrients and minerals from the infected hosts [10]. Free iron may generate damaging free radicals and therefore is typically maintained at low concentrations within the host. Bacterial pathogens are known to secrete extracellular proteins that lyse erythrocytes in order to acquire the free iron released from heme [11]. Hemolysins, one such class of secreted proteins, are exotoxins that have lytic activity against erythrocytes and are often implicated as virulence factors. These proteins also allow bacteria to evade the immune system by escaping from phagosomes [12]. Many hemolysins act by forming pores on the target cell’s membrane while others act through surfactant-like or enzymatic mechanisms [13].

Hemolysins have been implicated in a variety of complications arising from bacterial infections, ranging from intestinal tract disease to septicemia. Hemolysins produced by *Escherichia coli* have been associated with enterotoxicity and septicemia [14], whilst those secreted by *Vibrio vulnificus* [15], *Aeromonas hydrophila* [16] and *Yersinia ruckeri* [17] have been detected in high concentrations during fatal infections. Radke et al. [18] reported that *Listeria monocytogenes* listeriolysin disrupts lysosomal and phagosomal membranes to allow for bacterial escape into the host cytoplasm to initiate bacterial replication. Hemolysins have also been previously reported for members of the *B. cepacia* complex [19, 20] and it is known that different strains produced different hemolysins with differing functions. The Japanese clinical strain had been reported to produce a hemolysin called cepalysin that is capable of forming pores up to 30 nm in diameter [21]. Two separate hemolysins have been described for U.S clinical isolates with a size of 72 kDa and 22 kDa [22]. The hemolysins of British isolates are capable of inducing apoptosis and degranulation of mammalian phagocytes [19] and thus quite distinct from both the Japanese and the U.S strains due to its lack of pore forming capacity.

A number of studies have reported the expression of several *B. pseudomallei* heat-stable and heat-labile toxins that exhibited cytolytic and hemolytic activity [23–25]. Elevated levels of either cytotoxicity or hemolytic activity have also been detected in supernatant extracts of stationary growth phase cultures [24]. However, it is still unclear if this hemolytic phenotype is the result of a single enzyme or a result of the combined outcomes from the reactions of different proteins.

Open reading frames (ORFs) annotated as hypothetical proteins make up almost 25 % of the *B. pseudomallei* genome [26]. Cruz-Migoni et al. [7] reported a genome-wide project to characterize *B. pseudomallei* hypothetical proteins in an attempt to discover proteins with novel functions, which may include putative virulence factors. In this paper, we report the characterization of the protein encoded by the coding region bpsl1375 and provide definitive evidence of its ability to lyse erythrocytes. *In silico* analysis revealed sequence level similarities to members of the N-acyltransferase superfamily (Conserved Domains Database ID: cl00357) from the COG3176 group of the Clusters of Orthologous Groups (COGs) database. Members of COG3176 have previously been annotated as putative hemolysins.

## Results

### BPSL1375 is a member of the N-acyltransferase superfamily

A systematic approach to characterize the functions of coding regions annotated as hypothetical proteins by Holden et al. [27] resulted in the extraction of all such sequences followed by resubmission for sequence alignment based analysis. This included targeted searches for previously reported virulence and pathogenicity factors such as various hemolysins from other Gram negative bacteria. One of these searches identified motifs previously observed in the hemolysin of *Ralstonia eutropha* within the coding region of an ORF originally annotated as encoding the hypothetical protein BPSL1375 by Holden et al. [27]. A database search of the BPSL1375 sequence in GenBank revealed that the sequence contained domains that could be remotely associated with the N-acyltransferase (NAT) superfamily and shared sequence similarity to other proteins that were also annotated as hypothetical proteins. The NAT superfamily (Conserved Domains Database ID: cl00357) consists of various enzymes that characteristically catalyze the transfer of an acyl group onto its substrate. Among the known members of this superfamily are proteins that have been annotated as putative hemolysins (COG3176). The PATRIC database’s [28] entry for BPSL1375 has been updated to take into account this known homology to hemolysin and thus carries a revised annotation for BPSL1375 as ‘putative hemolysin’.

This homology to the NAT superfamily enabled us to refine our BLASTP database searches using the higher quality curated datasets in SWISSPROT/UniProt [29] and the Protein Data Bank (PDB) [30]. The UniProt search yielded 13 hits, all with scores lower than 40, E values of 0.07 or higher and sequence identities of 25 % and lower. Two of the hits were for acyl-homoserine-lactone (AHL) synthase, a well characterized member of the NAT superfamily. The BLASTP search against the PDB also yielded hits with scores lower than 30, E values of 1.7 or higher and sequence identities of 37 % or lower. Again, we were able to observe a hit for an acyl-homoserine-lactone synthase, specifically, the sequence for the crystal structure of *Pseudomonas aeruginosa* LasI [31] with the PDB code 1RO5.

**Fig. 1:**
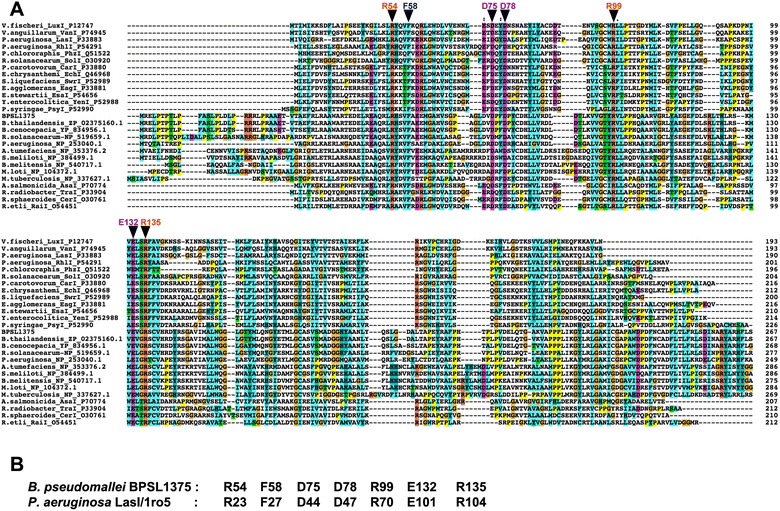
Multiple sequence alignment of BPSL1375 and corresponding residue numbers of BPSL135 and LasI/1ro5. Multiple sequence alignment has been done to identify highly conserved regions such as residues that may be potential active sites or binding motifs of BPSL1375 sequence against 26 other sequences from the COG3176 family and representatives from the lysophospholipid acyltransferase (LPLAT) family to which AHL synthase belongs. **a** Multiple sequence alignment of BPSL1375 with representatives of the COG3176 family and representatives of the LPLAT family including the LasI sequence (PDB crystallographic structure 1ro5) – each sequence is labeled as either Genus.species_Protein Name_Accesion number for Uniprot sourced data or Genus.species_Accession_number for GenBank sourced data; **b** Corresponding residue numbers in BPSL1375 and *P. aeruginosa* LasI/1ro5 for the seven residues conserved throughout the alignment

To identify highly conserved regions such as residues that may be potential active sites or binding motifs, we carried out multiple sequence alignment of the BPSL1375 sequence against 26 other sequences from the COG3176 family and representatives from the lysophospholipid acyltransferase (LPLAT) family to which AHL synthase belongs. From the alignments, we identified 7 residue positions – Arginine 54 (R54), Phenylalanine 58 (F58), Aspartic Acid 75 (D75), Aspartic Acid 78 (D78), Arginine 99 (R99), Glutamic Acid 132 (E132) and Arginine 135 (R135) that were conserved in all 27 sequences (Fig. 1a). The equivalent residue numbers for the LasI sequence are provided in Fig. 1b. Six of these residues (R54, D75, D78, R99, E132 and R135) carried charged side chains while F58 is a hydrophobic amino acid.

### BPSL1375 and its orthologs are conserved in *Burkholderia* spp.

**Fig. 2:**
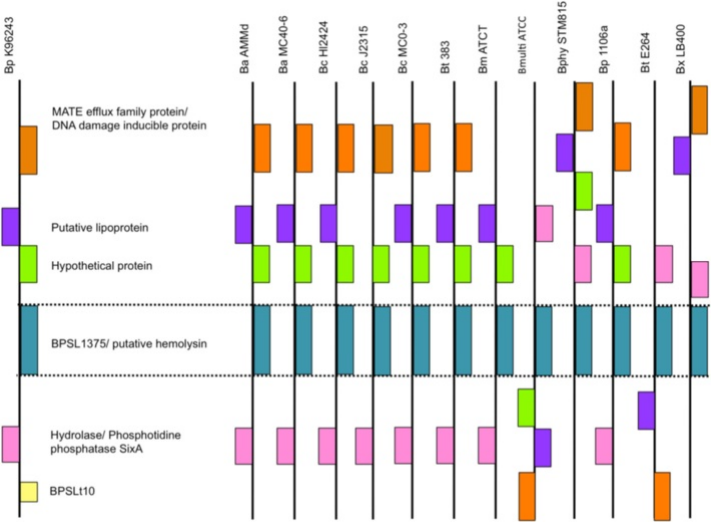
Conservation of *bpsl1375* and neighboring genes in other members of the *Burkholderia* genus. The conservation of bpsl1375 matched twelve orthologous sequence regions within the *Burkholderia* genus although seven of these appear to have possibly undergone various levels of rearrangement. Bp: *B. pseudomallei*; Ba: *B. ambifaria*; Bc: *B. cenopacia*; Bt: *B. thailandensis*; Bm: *B. mallei;* B. multi ATCC: *B. multivorans* ATCC17616; Bphy: *B. phymatum*; Bx: *B. xenovorans*

We then investigated whether BPSL1375 is common to all *Burkholderia* spp. and twelve orthologs were discovered in other *Burkholderia* species with available genome sequences. These *Burkholderia* spp. orthologs were mainly annotated as either hemolysin-like proteins or ornithine-acyl N-acyltransferases. We further explored this conservation by analyzing the presence and arrangement of the *bpsl1375* orthologs and the flanking coding regions in the other *Burkholderia* species. We were able to match twelve orthologous sequence regions within the *Burkholderia* genus although seven of these appear to have possibly undergone various levels of rearrangement (Fig. 2).

### Scanning electron microscopy reveals evidence of hemolysis by BPSL1375

**Fig. 3:**
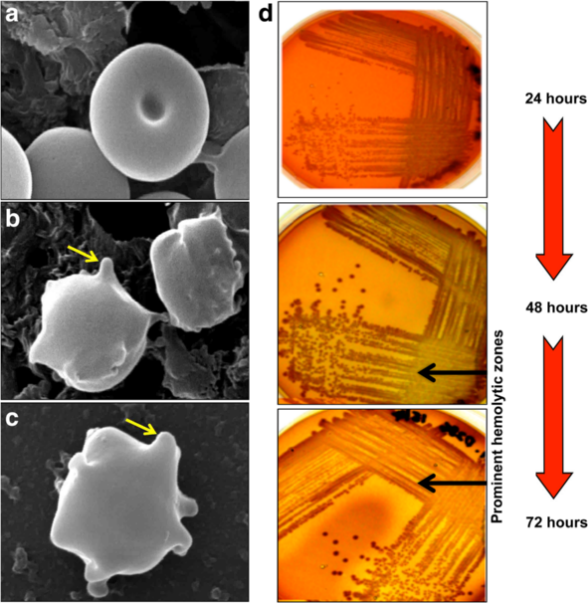
A comparison between the electron micrographs of cytoplasmic blebbing on the membrane and recombinant *E. coli* carrying the bpsl1375 gene grown on blood agar plates. Scanning electron microscopy revealed that sheep erythrocytes treated with the recombinant BPSL1375 displayed crenated shapes with cytoplasmic blebbing on the membrane (**a**) compared to untreated erythrocytes that presented the usual biconcave discs appearance (**b**), nevertheless, blebbing was not as notable as the positive control for lysis (**c**). Recombinant *E. coli* carrying the *bpsl1375* gene (see below) that were grown on blood agar plates also showed clear hemolytic zones (**d**). **a** Normal biconcave disc shape of untreated erythrocytes in PBS; **b** BPSL1375 treated erythrocytes showing the presence of discrete blebs (yellow arrow) on the erythrocyte; and **c** erythrocytes in sterile distilled water that also show the blebs (yellow arrow) as a positive control for lysis. **d** Recombinant *E. coli bpsl1375*colonies grown on trypticase soy agar with 5 % sheep blood after 24, 48 and 72 h of incubation at 37 °C showing clear hemolytic zones

The potential for BPSL1375 to have hemolytic activity as suggested by the sequence alignment was investigated by incubating purified recombinant BPSL1375 (Additional file 1) with sheep erythrocytes. The expression and purification of recombinant BPSL1375 is discussed in more detail below. Scanning electron microscopy (Fig. 3) revealed that sheep erythrocytes treated with the recombinant BPSL1375 displayed crenated shapes with cytoplasmic blebbing on the membrane (Fig. 3b) compared to untreated erythrocytes that presented the usual biconcave discs appearance (Fig. 3a), nevertheless, blebbing was not as notable as the positive control for lysis (Fig. 3c). Recombinant *E. coli* carrying the *bpsl1375* gene (see below) that were grown on blood agar plates also showed clear hemolytic zones (Fig. 3d). Confirmation of BPSL1375 hemolytic activity then led us to perform modeling and generation of recombinant mutants to enable the determination of key residue(s) required for hemolytic activity.

### Modeling and structural analysis

The importance of the seven conserved residues identified by multiple sequence alignment of BPSL1375 with other available AHL synthases was initially investigated using the crystal structure of *P. aeruginosa* LasI (PDB: 1RO5) as reference. The corresponding conserved residues of BPSL1375 in LasI are R54/R23, F58/F27, D75/D44, D78/D47, R99/R70, E132/E101 and R135/R104 (Fig. 1b). The structural study of 1RO5 indicated that these residues were involved in the formation of the substrate binding pocket in an electrostatic cluster of the molecule [31], thus suggesting that these residues play key roles in supporting the proper structure and formation of the binding site.

**Fig. 4:**
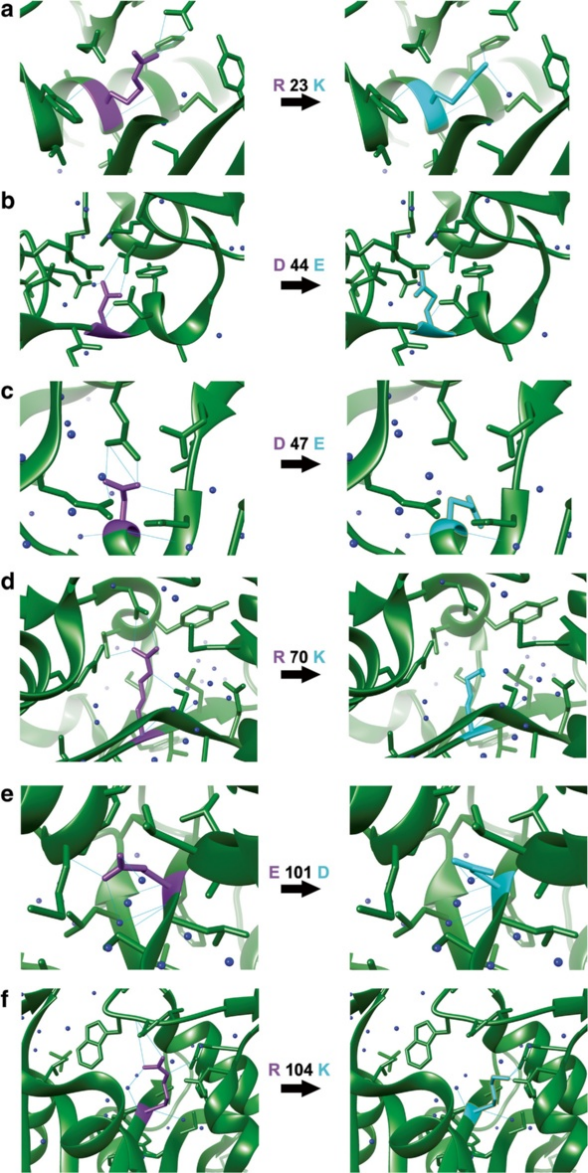
Models of the amino acid substitutions generated using Chimera. Substitutions of the different residues also resulted in changes in the number of hydrogen bonds with the neighboring residues. **a** R23K, **b** D44E, **c** D47E, **d** R70K, **e** E101D and **f** R104K

Each of the seven conserved residues was substituted *in silico* with residues from the same group of amino acids such as R with K and D with E in order to maintain the protein’s general fold as well as the local conformations at the substitution sites [32]. Visual analysis using Chimera molecular graphics [33] revealed the consistency of the local conformation for the substituted residues compared to the original residues indicating the suitability of the substitution in conserving the position and orientation of the original residue in 3D space (Fig. 4). We further investigated changes in the number of hydrogen bonds with the neighboring residues that resulted from the substitution. The substitution of R23K reduced the number of hydrogen bonds formed from 5 to 4 (Fig. 4a), D44E from 4 bonds to 3 (Fig. 4b), D47E from 4 bonds to 2 bonds (Fig. 4c), R70K from 5 bonds to 3 (Fig. 4d), E101D from 6 bonds to 5 bonds (Fig. 4e) and R104K with 5 bonds reduced to 3 bonds (Fig. 4f). With these mutant models as a guide, we expect the changes in the number of hydrogen bonds to change the interactions between the residues involved, thus, possibly affecting the overall catalytic properties of the molecule. In the next section, we demonstrate the effect of each substitution on the hemolytic activity of BPSL1375.

### Characterization of BPSL1375 and site directed mutants

In order to characterize the BPSL1375 protein, the *bpsl1375* gene was cloned from a clinical isolate (*B. pseudomallei* D286) available in our laboratory. The primers used for the work reported here (Table 1) were designed using the available *B. pseudomallei* K96243 genome sequence. The amplified 819 bp ORF of *bpsl1375*, encoding 272 amino acids, was successfully ligated into the expression vector pET200/D-TOPO. The 37 kDa BPSL1375 protein was expressed by *E. coli* BL21 (DE3) and purified by affinity chromatography.

**Table 1:** Primers used for PCR amplification of *bpsl1375* and site directed mutagenesis of *bpsl1375*

Primer name	Primer Sequence (5’-3’)
BPSL1375_F	CACCATGCGAGAACTGCCGA
BPSL1375_R	GCGCGGCAGCGGATCGCTCA
BPSL1375_R54K_F	GAAGCGCAGCGGCTGAAGCACAGTGTGTTCGCC
BPSL1375_R54K_R	GGCGAACACACTGTGCTTCAGCCGCTGCGCTTC
BPSL1375_D75E_F	GGCCTCGACGTCGAGCCGTTCGACC
BPSL1375_D75E_R	GGTCGAACGGCTCGACGTCGAGGCC
BPSL1375_D78E_F	GACGTCGATCCGTTCGAGCCGTACTGC
BPSL1375_D78E_R	GCAGTACGGCTCGAACGGATCGACGTC
BPSL1375_R99K_F	TGAAGGTCGTCGGCACCTATAAGGTGCTGCCGCC
BPSL1375_R99K_R	GGCGGCAGCACCTTATAGGTGCCGACGACCTTCA
BPSL1375_E132D_F	CGAAGATGGTCGATGTCGGCCGCTCGT
BPSL1375_E132D_R	ACGAGCGGCCGACATCGACCATCTTCG
BPSL1375_R135K_F	GATGGTCGAAGTCGGCAAGTCGTGCGTGCATCGCG
BPSL1375_R135K_R	CGCGATGCACGCACGACTTGCCGACTTCGACCATC

The *E. coli* clones expressing recombinant BPSL1375 produced clear hemolytic zones around the colonies on trypticase soy agar containing 5 % sheep’s blood (Fig. 3d). Quantification of BPSL1375 hemolytic activity on 3 % sheep blood suspension for 1 h at 37 °C revealed up to 26.9 % hemolysis. To investigate the contribution of the conserved amino acids towards the observed BPSL1375 hemolytic activity, site directed mutagenesis was performed on R54, F58, D75, D78, R99, E132 and R135. Of the seven conserved positions, six residues were successfully substituted: R54K, D75E, D78E, R99K, E132D and R135K. All six successful site directed substitutions were then confirmed by Sanger sequencing.

The R135K and R99K substitutions resulted in a complete loss of hemolytic activity indicating that these residues may be critical for the hemolytic activity observed for BPSL1375 (Table 2). The other three substitutions of E132D, D75E and R54K resulted in a non-significant decrease of hemolytic activity. The hemolysis activity of E132D was recorded at 22 % whereas D75E and R54K hemolysis were at 18 % and 14 % respectively. This implied that although these residues are conserved within the COG3176 family members and most likely important for BPSL1375 hemolytic activity, they may not be directly involved in the catalytic reaction or interactions associated with hemolysis and perhaps, have substrate binding roles associated to substrate specificity determination. We also noted that the hemolytic activity of the D78E mutant increased up to 2.3 fold higher than the wild-type (Table 2). For the work reported here, a single sampling to quantify hemolysis by BPSL1375 was conducted at 1 h. Future studies should investigate the hemolytic activity of BPSL1375 and the site directed mutants over time.

**Table 2:** Quantitation of hemolytic activity of the recombinant wild type and mutant BPSL1375 proteins

Protein / Control	Hemolysis Activity (%)^a^
Distilled water	100
*B. pseudomallei* D286 lysate	80
Wild type recombinant BPSL1375	27
Recombinant R54K	14
Recombinant D75E	18
Recombinant D78E	61
Recombinant R99K	7
Recombinant E132D	22
Recombinant R135K	5
PBS	9

^a^Incubation with erythrocytes for 60 min at 37 °C

### Hemolysis induced by BPSL1375 wild type and mutant proteins are temperature dependent

**Fig. 5:**
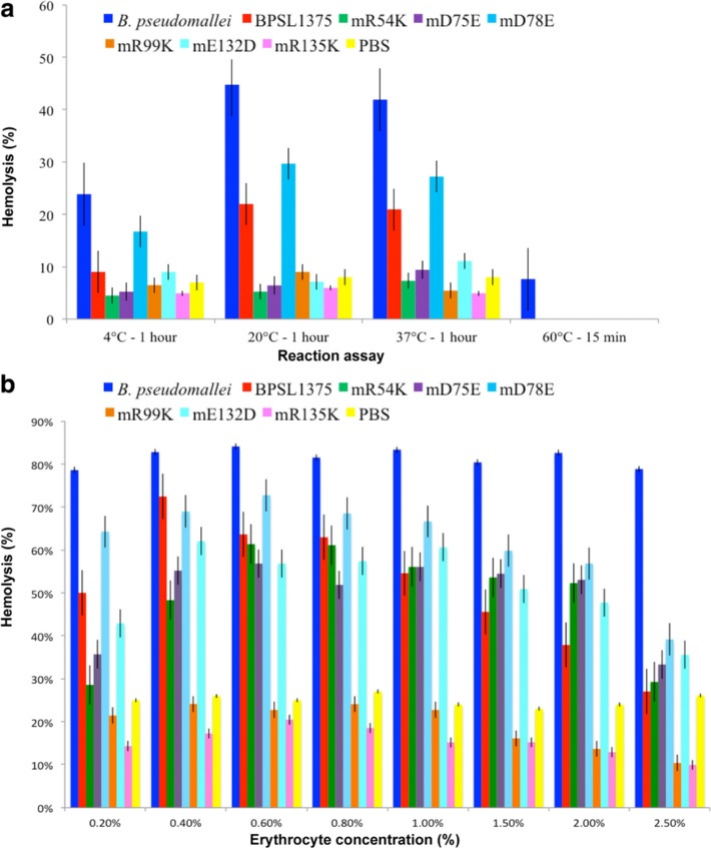
Hemolytic activity of the wild type and mutant proteins. The hemolytic activity was carried out on 1 % rabbit erythrocyte solution at different temperatures **a** and at different erythrocyte concentrations ranging from 0.2 to 2.5 % **b** after 60 min incubation at 37 °C. ‘*B. pseudomallei*’ refers to *B. pseudomallei* lysate;‘BPSL175’ refers to the recombinant protein; mutants are denoted by the scheme “m_Original residue_Residue number_Substituted residue”; PBS refers to phosphate buffered saline

The BPSL1375 recombinant wild type and mutant proteins were incubated individually in erythrocyte suspensions to observe hemolysis at four different temperatures; 4 °C, 20°, 37 °C and 60 °C (Fig. 5a). Incubation was fixed at 60 min for all reactions except for the 60 °C sample which was limited to 15 min. The mixtures were centrifuged and the level of hemoglobin released was measured at 545 nm. Poor hemolysis was observed for all samples at 4 °C, including the positive control *B. pseudomallei* lysate. However, hemolysis at 20 °C was the highest for *B. pseudomallei* lysate, BPSL1375, BPSL1375/D78E, BPSL1375/R99K and BPSL1375/R135K (Fig. 5a) whilst the highest hemolysis at 37 °C was noted for BPSL1375/E132D. Incubation at 60 °C abolished the activity of all samples except for *B. pseudomallei* lysate. The presence of a heat labile hemolysin has been reported for *B. pseudomallei* although it has not been specifically identified [23]. Our results suggest that BPSL1375 as wellas the mutants are heat-labile, and BPSL1375 may therefore be the previously uncharacterized heat labile hemolysin of *B. pseudomallei* first reported by Ashdown and Koehler [23].

### BPSL1375 is inhibited by high erythrocyte concentrations

The amount of hemolysin needed to lyse a single erythrocyte molecule was determined by incubating a fixed quantity of *B. pseudomallei* lysate, BPSL1375 and the mutant proteins with different concentrations (0.2 to 2.5 %) of erythrocytes. According to Oberley et al. [34], one molecule of hemolytic protein is sufficient for lysis and therefore the amount of hemoglobin released by a constant amount of protein is proportional to the erythrocyte concentration until a plateau is reached. Therefore, if more than one molecule of protein is required for lysis, the amount of hemoglobin released should increase to a peak and then decrease. After 1 h at 37 °C, the mixtures were centrifuged and the amount of hemoglobin released into the supernatant was read at 545 nm. The *B. pseudomallei* lysate lysed erythrocytes at all concentrations of sheep blood while for BPSL1375 and its mutants, the percentage of hemolysis decreased as erythrocyte concentrations increased (Fig. 5b).

These results suggest that BPSL1375 employs a multi-hit hemolytic mechanism that is similar to the hemolysins of other pathogenic bacteria such as *B. cereus* [35], *Gardenella vaginalis* [36], *Streptococcus suis* and *Synechoctus* sp. [37]. This mechanism is explained by the ability of a single toxin to bind only to a limited quantity of cells to induce hemolysis and is inhibited in the presence of a high ratio of erythrocytes to toxin molecule [38]. Therefore, keeping the hemolysin concentration constant and increasing the number of erythrocytes suggests that the optimal ratio is not achieved due to the presence of excess erythrocytes. For instance, Inoue et al. [38] described that two to five molecules of streptolysin O and *Clostridium perfringens* delta toxin are required per erythrocyte to induce hemolysis. This characteristic was maintained in BPSL1375 and all of the mutants where similar reduction in hemolytic activity was observed at higher erythrocyte concentrations.

## Discussion

Our characterization of the *B. pseudomallei* protein BPSL1375 that was originally annotated as a hypothetical protein by Holden et al. [27] but subsequently revised to ‘putative hemolysin’ in the PATRIC database, has enabled this protein to be classified as a member of the N-acyltransferase (NAT) superfamily that includes several putative hemolysins, other hypothetical proteins and AHL synthases - namely LasI of *Pseudomonas aeruginosa*. The BPSL1375 sequence can be further grouped into the COG3176 cluster with members that contain hemolysin-like domains. This hemolytic potential was validated by hemolysis assays and site directed mutants. The hemolytic activity demonstrated by the expression of BPSL1375 (Fig. 3d) is evidence that the protein is the actual structural hemolysin and that BPSL1375 is not merely a regulator of hemolytic activity. To our knowledge, this is the first member of the NAT superfamily with experimental evidence of hemolytic activity.

Multiple sequence analysis to discover BPSL1375 amino acids that were shared with its homologs revealed seven conserved residues (R54, F58, D75, D78, R99, E132 and R135) that are likely important in the protein’s function. Cross-referencing of these residues with the observations reported by Gould et al. [31] for the LasI crystal structure revealed that these residues are known to be crucial for the functionality of LasI. These equivalent residues in LasI were deemed to be essential for AHL synthesis and contribute towards the stability of intra-structural interactions with possible catalytic functions. Additionally, the equivalent residue for E101 (corresponding to E132 in BPSL1375) is bound to two commonly conserved water molecules in AHL synthase that is known to partake in the enzyme’s catalytic mechanism [31].

Based on our modeling of the LasI structure (PDB ID: 1RO5), the most suitable amino acid substitutions that preserved the function of the conserved residues were R54K, F58W, D75E, D78E, R99K, E132D and R135K. These substitute amino acids were selected because they can maintain a similar 3D space and amino acid sequence characteristic to that of the wild type protein. The substitution of R with K and D with E were carried out to minimize the disturbance of protein fold and structural characteristics, either locally or globally. In this study, the substituted residues were visually scrutinized using Chimera to show the reduction and addition of hydrogen bonds that resulted from the changes, suggesting that the resulting mutants of these residues may show different levels of hemolytic activity.

Consequently, site directed mutagenesis of these seven conserved residues resulted in six successful substitutions and each mutant displayed different degrees of hemolytic capacity. The mutants R99K and R135K showed a complete loss of hemolytic activity and interestingly the D78E mutant showed a significant 2.3 fold increase in hemolytic activity. Although the exact molecular mechanisms for the hemolysis we observed is not yet known, the differences between the degree of hemolysis for each mutant can provide important clues with regards to the contribution of these residues in molecular interactions that result in hemolytic activity. The complete loss of hemolytic activity for mutants of R99 and R135 highlighted that these residues must play a crucial role in the activity of BPSL1375.

Our investigations into the hemolytic activity of BPSL1375 and the six mutants on different erythrocyte concentrations revealed that hemolytic activity decreased with an increase in the ratio between the protein molecule and erythrocyte thus implying that one erythrocyte required a certain number of ‘hits’ by the protein to induce lysis, a mechanism known as ‘multi-hits’. This mechanism has also been observed in *Synechocystic* sp. hemolysin, a cyanobacterial toxin [37].

## Conclusions

In this study, a *B. pseudomallei* open reading frame identified using the BpK96243 genome sequence [27] that was initially annotated to encode a hypothetical protein, was characterized as an N-acyltransferase that exhibits hemolytic activity. Genetic manipulation of the residues associated with N-acyltransferase activity resulted in marked detectable variations of hemolytic activity. Although the exact mechanism that resulted in the differences in the exhibited hemolytic activity could not be pinpointed, this study demonstrated the highly useful utility that modeling of amino acid substitutions of available well characterized high resolution structures in the PDB can be reliably transferred to a homolog with very low sequence similarity and thus enabling functional characterization of the remote homolog. Based on our results, R99 and R135 are most likely crucial for function due to the complete loss of activity resulting from their substitution and therefore likely to be among the most highly conserved residues for this family of hemolytic N-acyltansferase (COG3176 family). Our work also demonstrated that BPSL1375 and the mutants possess a multi-hit hemolytic activity towards erythrocytes and that this N-acyltransferase enzyme is not heat stable.

## Methods

### Sequence analysis

Sequence homologs of BPSL1375 were retrieved using BLASTP searches (http://blast.ncbi.nlm.nih.gov) against the following databases: GenBank refseq, SWISSPROT/UniProt [29], Protein Data Bank (PDB) [30] and the *Burkholderia* Genome Database [39]. Multiple sequence alignments were performed with ClustalW [40].

### Protein structure modeling and analysis

Models were visualized and analyzed using Chimera [33]. Modeling of mutations, structure comparisons and model refinements were also carried out using Chimera

### Strains, vectors, enzymes and chemicals

*B. pseudomallei* strain D286 was previously isolated from a fatal melioidosis case at Kuala Lumpur Hospital [41]. A stock culture of this strain was obtained from the Pathogen Laboratory, Faculty of Science and Technology, Universiti Kebangsaan Malaysia. *E. coli* BL21(DE3) and pET200/D-TOPO were obtained from Invitrogen, USA and other kits as well as reagents were obtained from standard commercial resources.

### Primer design

The ORFs for *B. pseudomallei* K96243 were generated using ARTEMIS 13.0. Corresponding primers were then designed for the ORF identified as bpsl1375 (Table 1). An additional CACC sequence (5’-CACCATGCGAGAACTGCCGA-3’) was added to the 5’- end to allow direct cloning into the expression vector. The primers (Table 1) for Quik-Change site directed mutagenesis were designed using a program provided by Stratagene.

### Genomic DNA preparation

*B. pseudomallei* D286 was plated on Ashdown selective agar plates. A single colony was inoculated into Brain Heart Infusion broth (Pronadisa, Spain) and grown overnight at 37 °C; the bacterial cells were harvested and the cell pellet was dissolved in TE buffer (10 mM Tris–HCl, 1 mM EDTA, pH 7.5). The pellet was treated with Proteinase K (20 mg/ml) and incubated at 37 °C for 1 h. CTAB-NaCl (5 M NaCl) (Sigma, USA) was added and the incubation was continued at 65 °C for 20 min. *B. pseudomallei* genomic DNA extraction was carried out according to standard protocols as described by Richards et al. [42]. Purification of genomic DNA was carried out according to the standard phenol chloroform method and precipitated using 0.6 volume of isopropanol.

### Amplification and cloning of *bpsl1375*

DNA amplification by polymerase chain reaction (PCR) was carried out in a final volume of 50 μl containing 10 X Expand High Fidelity buffer with 15 mM MgCl_2_ (Sigma Chemical, USA), 10 mM dNTP (Sigma Chemical, USA), 200 ng/μl *B. pseudomallei* genome, 25 μM forward and reverse primers, 3.5 U/μl High Fidelity *Taq* polymerase (Roche, German) and ddH_2_O. The amplification was performed over 30 cycles of denaturation at 94 °C for 1 min, annealing at 67 °C for 1 min and elongation at 72 °C for 1 min. A final extension step was carried out for 10 min at 72 °C. The amplified products were cloned into pET200/D-TOPO according to the manufacturer’s recommendation (Invitrogen, USA). The gene orientation and sequence fidelity were verified by Sanger DNA sequencing.

### Construction of mutants

Single residue mutations were produced using the Stratagene QuikChange site-directed mutagenesis kit. The wild type pET200-bpsl1375 construct was used as a DNA template. The mutations were confirmed by automated DNA sequencing. Six mutants were produced: R54K, D75E, D78E, R99K, E132D and R135K.

### Expression and purification of BPSL1375 and its related mutants

Recombinant bpsl1375 and the corresponding mutants were transformed into E. coli BL21 Star (DE3) according to the kit manufacturer’s recommendations (Invitrogen, USA). The bacterial cells were grown on LB (Pronadisa, Spain)-Kanamycin (30 μg/ml) agar plates for 16 h at 37 °C. A single colony was inoculated into 5 ml LB-Kanamycin (30 μg/ml) and incubated at 30 °C overnight with shaking at 250 rpm. The overnight culture was subcultured into 50 ml of LB-Kanamycin (30 μg/ml) and grown at 30 °C with shaking at 250 rpm. At OD_600_ = 0.5, expression was induced with 1 mM IPTG (Promega, USA) for 6 h at 30 °C. Cells were harvested and protein extraction procedures were performed at 4 °C. The cell pellet was resuspended in 1 mL PBS buffer, sonicated for 10 s at 50 Hz and then centrifuged at 3200 g for 30 min. The supernatant containing soluble protein was collected and stored at −20 °C. His-tagged protein purification was performed using a HisTrap ^TM^ HP column (GE Healthcare). The column was washed with wash buffer (50 mM NaH_2_PO_4_, 300 mM NaCl, 50 mM imidazole, pH 7.4) and eluted with elution buffer (50 mM NaH_2_PO_4_, 300 mM NaCl, 300 mM imidazole, pH 7.4). The eluted protein fractions were concentrated using Vivaspin 6 (MW 10 kDa) (Vivascience, Germany). Protein concentration was determined using the bicinchoninic acid method (Sigma Chemical, USA) and analyzed by 12 % SDS-PAGE electrophoresis followed by Western immunoblotting with anti-mouse IgG conjugated with HRP (Invitrogen, USA). C

### Ethics statement and consent

Blood samples from rabbit and sheep were generously provided by the Animal House Facility, Universiti Kebangsaan Malaysia (UKM). Blood was drawn in accordance with the UKM animal ethics guideline formulated based on the guidelines of the National Health and Medical Research Council of Australia. The experiments were approved by the UKM Animal Ethics Committee (UKMAEC) under approval number FST/SBB/2010/SHEILA/24-AUGUST/320. All animals were maintained in a positive pressure environment at 20–25 °C, subjected to a 12 h light/dark cycle and fed with a protein-enriched diet and water *ad libitum*. No human subjects were used in the experiments reported in this paper.

### Preparation of rabbit erythrocyte suspensions

Rabbit whole blood in anticoagulant citrate dextrose was centrifuged at 750 *g* for 10 min. Plasma and white blood cells were discarded by removing the supernatant. Erythrocytes were washed with PBS three times following centrifugation at 750 *g* for 10 min.

### Measurement of hemolytic activity

Rabbit erythrocyte suspensions were adjusted to 4 % with PBS. The purified proteins and *B. pseudomallei* lysates were adjusted to 10 μg/ml. Then, 0.1 ml of this solution was incubated with 0.1 ml of 4 % erythrocyte suspension at 37 °C for 1 h. The samples were centrifuged at 750 *g* for 5 min to remove undamaged erythrocytes. The concentration of released hemoglobin was estimated by reading the absorbance at 545 nm against the control background lysis solution (erythrocyte suspension with PBS) using the Ultraspec 2100 pro (Amersham Biosciences). The percentage of hemolysis was calculated by normalizing against the absorbance of a mixture of erythrocytes and distilled water at the same ratio, which showed complete hemolysis.

To determine the effect of hemolytic activity on different amounts of red blood cells, rabbit erythrocyte suspensions at different concentrations (0.2 %, 0.4 %, 0.6 %, 0.8 %, 1.0 %, 1.5 %, 2.0 % and 2.5 %) in PBS were used. These diluted cell suspensions were incubated with an equal volume (1.0 μg) of *B. pseudomallei* lysate, purified BPSL1375 or the mutant protein for 1 h at 37 °C and then centrifuged at 750 *g* for 5 min to determine the hemolytic activity. For temperature-dependent studies, equal volumes (1.0 μg) of *B. pseudomallei* lysate, purified BPSL1375 and the mutant proteins were incubated with 1 % rabbit erythrocytes at 4 °C, 20 °C and 37 °C for 1 h and 60 °C for 15 min. The mixtures were centrifuged at 750 *g* for 5 min and the residual hemolytic activity of the supernatant was determined.

### Scanning electron microscopy

Sheep erythrocytes (5 %) incubated with BPSL1375 were fixed in 2 % (v/v) glutaraldehyde, 0.1 M PBS (pH 7.4) overnight. Prior to dehydration in ethanol, samples were washed in PBS three times for 10 min. Critical point drying was performed with acetone and sputter-coated with gold-palladium. The samples were observed with a Philips XL30 scanning electron microscope at the Universiti Kebangsaan Malaysia Electron Microscopy Facility.

## Additional file

Additional file 1:
**Figure A and B.** SDS PAGE (A) of soluble fractions for wild type recombinant BPSL1375, R54K mutant, D75E mutant and the corresponding Western blot (B). Lane 1: Broad range marker; Lane 2: E. coli BL21 (DE3) StarTM - IPTG; Lane 3: E. coli BL21 (DE3) StarTM + IPTG; Lane 4: BPSL1375 – IPTG; Lane 5: BPSL1375 + IPTG; Lane 6: R54K – IPTG; Lane 7: R54K + IPTG; Lane 8: D75E – IPTG; Lane 9: D75E + IPTG; Lane 10: purified BPSL1375. **Figure C and D.** SDS PAGE (C) of soluble fractions for wild type recombinant BPSL1375, D78E mutant, R99K mutant and the corresponding Western blot (D). Lane 1: Broad range marker; Lane 2: E. coli BL21 (DE3) StarTM - IPTG; Lane 3: E. coli BL21 (DE3) StarTM + IPTG; Lane 4: BPSL1375 – IPTG; Lane 5: BPSL1375 + IPTG; Lane 6: D78E – IPTG; Lane 7: D78E + IPTG; Lane 8: R99K – IPTG; Lane 9: R99K + IPTG; Lane 10: purified BPSL1375. **Figure E and F.** SDS PAGE (E) of soluble fractions for wild type recombinant BPSL1375, E132D mutant, R135K mutant and the corresponding Western blot (F). Lane 1: Broad range marker; Lane 2: E. coli BL21 (DE3) StarTM - IPTG; Lane 3: E. coli BL21 (DE3) StarTM + IPTG; Lane 4: BPSL1375 – IPTG; Lane 5: BPSL1375 + IPTG; Lane 6: E132D – IPTG; Lane 7: E132D + IPTG; Lane 8: R135K – IPTG; Lane 9: R135K + IPTG; Lane 10: purified BPSL1375. (PDF 469 kb)
