# Supplementary material for: Characterization of Burkholderia pseudomallei protein BPSL1375 validates the Putative hemolytic activity of the COG3176 N-Acyltransferase family
Source: BMC Microbiol. 2015 Nov 23;15:270. doi: 10.1186/s12866-015-0604-4 (PMC4657338; doi:10.1186/s12866-015-0604-4)
Supplement: Additional file 1: — Figure A and B. SDS PAGE (A) of soluble fractions for wild type recombinant BPSL1375, R54K mutant, D75E mutant and the corresponding Western blot (B). Lane 1: Broad range marker; Lane 2: E. coli BL21 (DE3) StarTM - IPTG; Lane 3: E. coli BL21 (DE3) StarTM + IPTG; Lane 4: BPSL1375 – IPTG; Lane 5: BPSL1375 + IPTG; Lane 6: R54K – IPTG; Lane 7: R54K + IPTG; Lane 8: D75E – IPTG; Lane 9: D75E + IPTG; Lane 10: purified BPSL1375. Figure C and D. SDS PAGE (C) of soluble fractions for wild type recombinant BPSL1375, D78E mutant, R99K mutant and the corresponding Western blot (D). Lane 1: Broad range marker; Lane 2: E. coli BL21 (DE3) StarTM - IPTG; Lane 3: E. coli BL21 (DE3) StarTM + IPTG; Lane 4: BPSL1375 – IPTG; Lane 5: BPSL1375 + IPTG; Lane 6: D78E – IPTG; Lane 7: D78E + IPTG; Lane 8: R99K – IPTG; Lane 9: R99K + IPTG; Lane 10: purified BPSL1375. Figure E and F. SDS PAGE (E) of soluble fractions for wild type recombinant BPSL1375, E132D mutant, R135K mutant and the corresponding Western blot (F). Lane 1: Broad range marker; Lane 2: E. coli BL21 (DE3) StarTM - IPTG; Lane 3: E. coli BL21 (DE3) StarTM + IPTG; Lane 4: BPSL1375 – IPTG; Lane 5: BPSL1375 + IPTG; Lane 6: E132D – IPTG; Lane 7: E132D + IPTG; Lane 8: R135K – IPTG; Lane 9: R135K + IPTG; Lane 10: purified BPSL1375. (PDF 469 kb) [file 12866_2015_604_MOESM1_ESM.pdf]

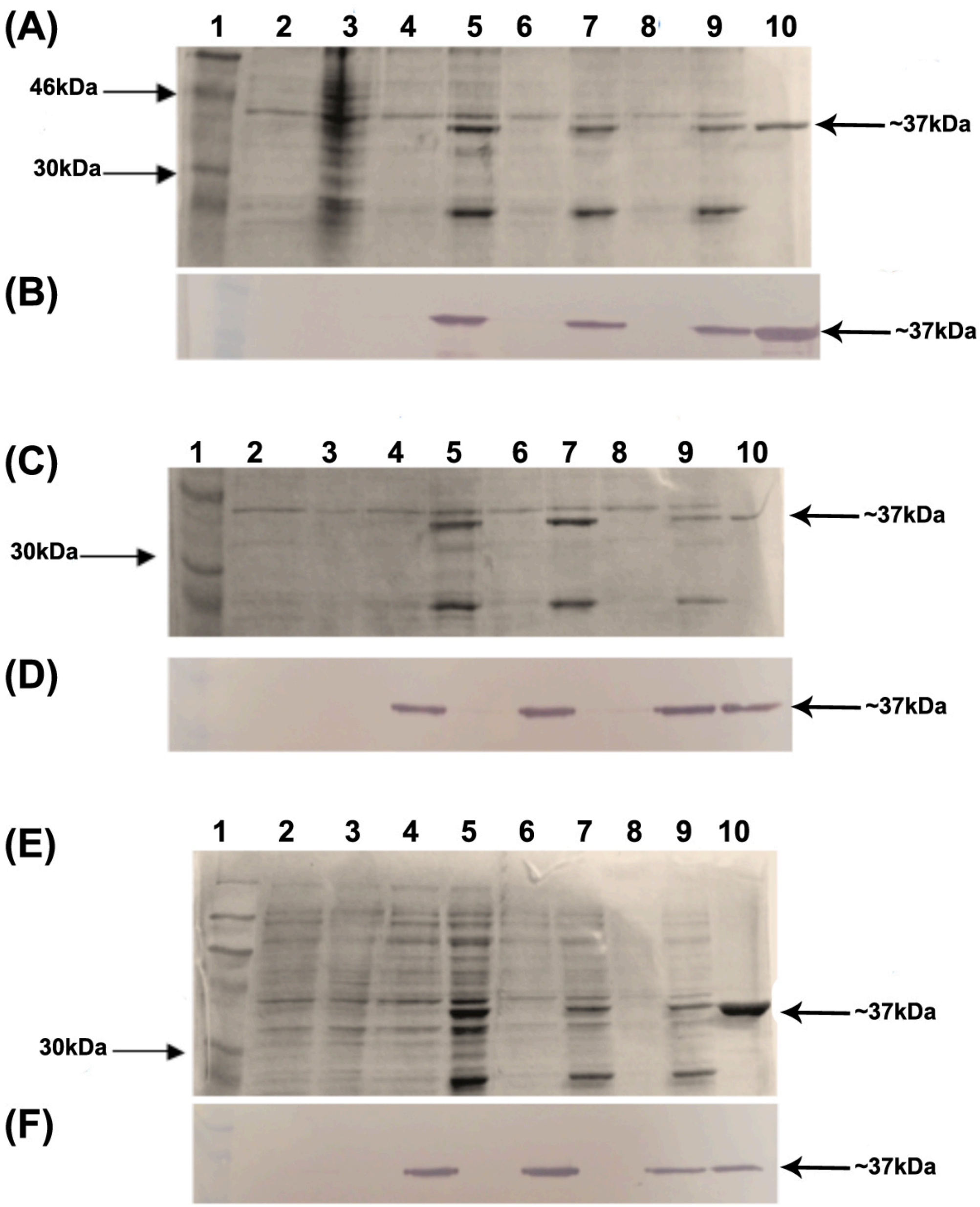

Additional File 1 Figure:

Figure A and B. SDS PAGE (A) of soluble fractions for wild type recombinant BPSL1375, R54K mutant, D75E mutant and the corresponding Western blot (B). Lane 1: Broad range marker; Lane 2: *E. coli* BL21 (DE3) StarTM - IPTG; Lane 3: *E. coli* BL21 (DE3) StarTM + IPTG; Lane 4: BPSL1375 – IPTG; Lane 5: BPSL1375 + IPTG; Lane 6: R54K – IPTG; Lane 7: R54K + IPTG; Lane 8: D75E – IPTG; Lane 9: D75E + IPTG; Lane 10: purified BPSL1375

Figure C and D. SDS PAGE (C) of soluble fractions for wild type recombinant BPSL1375, D78E mutant, R99K mutant and the corresponding Western blot (D). Lane 1: Broad range marker; Lane 2: *E. coli* BL21 (DE3) StarTM - IPTG; Lane 3: *E. coli* BL21 (DE3) StarTM + IPTG; Lane 4: BPSL1375 – IPTG; Lane 5: BPSL1375 + IPTG; Lane 6: D78E – IPTG; Lane 7: D78E + IPTG; Lane 8: R99K – IPTG; Lane 9: R99K + IPTG; Lane 10: purified BPSL1375.

Figure E and F. SDS PAGE (E) of soluble fractions for wild type recombinant BPSL1375, E132D mutant, R135K mutant and the corresponding Western blot (F). Lane 1: Broad range marker; Lane 2: *E. coli* BL21 (DE3) StarTM - IPTG; Lane 3: *E. coli* BL21 (DE3) StarTM + IPTG; Lane 4: BPSL1375 – IPTG; Lane 5: BPSL1375 + IPTG; Lane 6: E132D – IPTG; Lane 7: E132D + IPTG; Lane 8: R135K – IPTG; Lane 9: R135K + IPTG; Lane 10: purified BPSL1375.
